# Speed and Agility Predictors among Adolescent Male Football Players

**DOI:** 10.3390/ijerph19052856

**Published:** 2022-03-01

**Authors:** Cíntia França, Élvio Gouveia, Romualdo Caldeira, Adilson Marques, João Martins, Helder Lopes, Ricardo Henriques, Andreas Ihle

**Affiliations:** 1 Department of Physical Education and Sport, University of Madeira, 9020-105 Funchal, Portugal; erubiog@uma.pt (C.F.); romualdo.caldeira@gmail.com (R.C.); hlopes@staff.uma.pt (H.L.); 2 LARSYS, Interactive Technologies Institute, 9020-105 Funchal, Portugal; 3 Center for the Interdisciplinary Study of Gerontology and Vulnerability, University of Geneva, 1205 Geneva, Switzerland; andreas.ihle@unige.ch; 4 CIPER, Faculty of Human Kinetics, University of Lisbon, 1499-002 Lisbon, Portugal; adncmpt@gmail.com (A.M.); jfigueiramartins@gmail.com (J.M.); 5 ISAMB, University of Lisbon, 1499-002 Lisbon, Portugal; 6 Centro de Estudos de Educação, Faculdade de Motricidade Humana e UIDEF, Instituto de Educação, Universidade de Lisboa, 1649-004 Lisboa, Portugal; 7 Centre for Tourism Research, Development and Innovation, University of Madeira, 9004-509 Funchal, Portugal; 8 Marítimo da Madeira—Futebol, SAD, 9020-208 Funchal, Portugal; ricardo.henriquesfut@gmail.com; 9 Swiss National Centre of Competence in Research LIVES—Overcoming Vulnerability: Life Course Perspectives, 1015 Lausanne, Switzerland; 10 Department of Psychology, University of Geneva, 1205 Geneva, Switzerland

**Keywords:** explosive strength, countermovement jump, squat jump, body fat, body composition

## Abstract

This study aimed to examine the associations between lower-body explosive strength (squat jump—SJ, and countermovement jump—CMJ), speed (10- and 35-m linear sprints), and agility (*t*-test) capacities, after controlling for crucial predictors such as chronological age (CA) and body composition. The sample was composed of 164 adolescent male football players from under 19, under 17, and under 15 age groups. Body fat percentage (BF%) was significantly and positively related to body mass, speed, and agility. In contrast, BF% was a significant negative predictor of lower-body explosive strength. Sprint and *t*-test times were significantly and negatively correlated with lower-body explosive strength. After controlling for CA and body composition, SJ was the most significant predictor, accounting for 36 to 37% of the variance observed in the 35 m linear sprint and the *t*-test performance. Our main results suggest that lower-body explosive strength, particularly in the SJ, is a significant predictor of male adolescent male football players’ speed and agility capacities. Conversely, detrimental relationships between BF% and these outcomes were observed. Sports agents should consider lower-body explosive strength development as part of the youth football training process, particularly to improve maximal sprint and change of direction times, which are crucial to game performance.

## 1. Introduction

Worldwide, football sports agents and coaches have relied on assessing players’ physical attributes in the processes of selection and talent identification [1]. Commonly, a particular emphasis has been given to lower-body explosive strength due to its close relationship with game performance, mainly through speed and agility capacities [2,3,4]. Overall, the sports literature mentions that players’ ability to produce force quickly is fundamentally necessary for achieving high-quality performance [5,6].

Previous research has described short accelerations and linear sprints as the most important actions during a football game since they frequently precede goals and other decisive actions [7]. Professional football players have become faster over time. Both speed and agility capacities are shown to distinguish groups from different performance levels [8]. The importance of speed and agility is reinforced by many studies conducted to identify effective conditioning programs to improve those attributes in football players [9,10,11]. Consistently, the optimization of lower-body explosive strength has been indicated as crucial to enhance sprinting and change of direction performance [3,10,12]. In a study on professional football players aged 18.3 ± 1.2 years, the authors reported that changes in the one-repetition maximum (1 RM) of the squat strength and in the 1 RM of the back squat were significantly and positively related to short sprint times at 5 m (pre-test: 1.11 ± 0.04 s, post-test: 1.05 ± 0.03 s), 10 m (pre-test: 1.83 ± 0.05 s, post-test: 1.78 ± 0.05 s), and 20 m (pre-test: 3.09 ± 0.07 s, post-test: 3.05 ± 0.05 s) [12]. In another study among adolescent male football players aged 16.2 ± 0.6 years, eight weeks of additional strength training with heavy loading of the lower limbs (70–90% 1 RM), resulted in significant changes in sprinting and agility tests [10]. Additionally, both the squat jump (SJ) and the countermovement jump (CMJ) performance improved in the experimental group (SJ pre-test: 0.36 ± 0.03 m, SJ post-test: 0.43 ± 0.02 m, CMJ pre-test: 0.37 ± 0.05 m, CMJ post-test: 0.42 ± 0.04 m) [10]. Indeed, previous literature has reported that strength training of lower limbs may potentiate SJ and CMJ performance [13,14].

Therefore, the SJ and the CMJ tests are frequently used by sports researchers to assess lower-body explosive strength [15,16]. Besides the potential to be used to evaluate explosive strength, both can also reveal muscular asymmetries or deficits in lower limbs [15]. Among adolescent football players, moderate to strong correlations have been reported between the SJ and CMJ, linear sprinting, and change of direction performance [3,17,18]. However, both chronological age (CA) and body size correlated significantly with vertical jumping [17,19,20]. In an adult population of both sexes, authors described that body fat (BF%) explained more variability in CMJ than any other single anthropometric variable [20]. In another study among youth football players, the main findings indicated that the jumping and sprinting profiles were largely correlated with both chronological age and maturity offset regardless of the influence of body size and training experience [17]. In addition, in a sample of youngsters, authors described greater performances in the CMJ associated with older ages, both in male and female participants [19]. Therefore, the lack of control of CA and BF% may lead to inaccurate assessments. For that reason, although previous research has been developed to analyze relationship between players’ physical attributes and lower-body explosive strength performance at several age ranges [21,22,23], it seems crucial to explore that relationship considering the influence of CA and BF%. This knowledge is of great interest to sports agents and coaches, particularly to optimize and select training programs that may enhance players’ long-term development.

Therefore, this study aimed to examine the associations between lower-body explosive strength, speed, and agility capacities after controlling for important confounders such as CA and body composition in a large sample of adolescent male football players. After controlling for CA and body composition variables, we hypothesized that superior performance in the lower-body explosive strength tasks (CMJ and SJ) would correspond to lower time spent in sprinting and change of direction tests.

## 2. Materials and Methods

### 2.1. Participants

One hundred and sixty-four male football players from under 19 (U19), under 17 (U17), and under 15 (U15) age groups participated in this study. Fifty-one players were U19 (age = 17.8 ± 1.1 years, height = 174.8 ± 6.1 cm, body mass = 68.8 ± 6.5 kg, body mass index = 22.4 ± 1.7 kg/m^2^); 62 players were U17 (age = 15.9 ± 0.6 years, height = 172.0 ± 7.3 cm, body mass = 64.5 ± 9.1 kg, body mass index = 21.8 ± 2.1 kg/m^2^); and 51 players were U15 (age = 14.0 ± 0.6 years, height = 165.5 ± 9.3 cm, body mass = 56.9 ± 10.5 kg, body mass index = 21.0 ± 2.9 kg/m^2^). All participants were enrolled in a regional competition in Portugal.

All procedures applied were approved by the Ethics Committee of the Faculty of Human Kinetics, CEIFMH N° 34/2021. The investigation was conducted following the Declaration of Helsinki, and informed consent was obtained from the participants or their legal guardians in the case of underage participants.

### 2.2. Anthropometric Characteristics

For the anthropometric measurements, participants were barefoot and only wearing shorts. Height was measured to the nearest 0.01 cm using a stadiometer (SECA 213, Hamburg, Germany). Body mass was measured to the nearest 0.1 kg using a portable scale (SECA 760, Hamburg, Germany). Skinfold thickness was measured to the nearest 0.1 mm at seven sites (biceps, triceps, subscapular, suprailiac, abdominal, thigh, and calf) using a skinfold caliper (Harpenden Skinfold Caliper, West Sussex, UK). Four evaluators performed the assessments. This team was composed of 4 sports professionals with considerable experience in anthropometric evaluation. Before the assessments, over the course of one week (6 h), training sessions for all the evaluators were conducted. The entire protocol was revised and discussed in theoretical–practical sessions. The skinfold caliper used in the Marítimo Training Lab was used following the calibration protocol recommended by the brand. Before each assessment, the skinfold calipers were checked. For all assessments, measurements were performed twice, and a third measurement was carried out in case of excessive difference. The scores of the two closest measures were averaged to reduce measurement error. All measurements were taken following the ISAK (International Society for the Advancement of Kinanthropometry) guidelines [24].

To estimate BF%, the equations proposed by Weststrate and Durenberg [25] and Siri [26] were used. First, body density was calculated for male individuals according to their CA. For pubertal individuals (13.8 ± 0.21 years), the following equation was applied: *d =* 1.0555 − 0.0352 (LOG sum of 4 skinfolds*) + 3.8 (age 10^−3^). The following equation was used for post-pubertal individuals (17.5 ± 0.39 years): *d =* 1.1324 − 0.0429 (LOG sum of 4 skinfolds*).

*sum of 4 *skinfolds*: *biceps* + *triceps* + *subscapular* + *suprailiac*

After calculating body density, the Siri equation [26] was used to estimate BF%: BF% = (4.95/D) − 4.5) × 100.

### 2.3. Static Strength

The handgrip protocol consisted of three alternated data collection trials for each arm, performed using a hand dynamometer (Jamar Plus+, Chicago, IL, USA). Participants were instructed to hold a dynamometer in one hand, laterally to the trunk with the elbow at a 90° position [27]. From this position, participants were instructed to squeeze as hard as possible and progressively and continuously squeeze the hand dynamometer for about two seconds. The dynamometer could not contact the participant’s body; otherwise, the trial was repeated. The best score of the three trials was retained for analysis.

### 2.4. Muscular Strength and Endurance

A sit-up protocol consisted of performing the greatest number of repetitions during 30 s [28]. Participants were instructed to start in a sitting position, torso vertical, hands behind their neck, bent knees (at 90 degrees), and feet on the floor. From this position, participants were instructed to stretch out on their back, shoulders in contact with the floor, then straighten up to a sitting position, bringing their elbows forward to make contact with their knees and/or pass them through their knees. Counting took place the moment the elbows touched or passed the knees. The absence of counting meant that the repetition had not been correctly performed. The total number of repetitions was used as the test score.

### 2.5. Lower-Body Explosive Strength

The squat jump (SJ) and the countermovement jump (CMJ) were applied to assess lower-body explosive strength [16]. Both protocols included four data collection trials and were performed using the Optojump Next (Microgate, Bolzano, Italy) system of analysis and measurement. In both tests, participants were encouraged to jump for maximum height. Before data collection, three experimental trials were performed by each participant to ensure correct execution.

The SJ protocol testing began with the participant in a squat position at a self-selected depth of approximately 90° of knee flexion, holding this position for the researchers’ count of three before jumping. If a dipping movement of the hips was evident, then the trial was repeated. The participants reset to the starting position after each jump.

In the CMJ protocol, participants began in a tall standing position, with feet placed hip-width to shoulder-width apart. Then, participants dropped into the countermovement position to a self-selected depth, followed by a maximal-effort vertical jump. Hands remained on the hips for the entire movement to eliminate any influence of arm swing. If the hands were removed from the hips at any point, or excessive knee flexion was exhibited during the countermovement, the trial was repeated. The participants reset to the starting position after each jump.

### 2.6. Speed

Linear speed was assessed with maximal sprints at 10 and 35 m, starting from a stationary position. Sprint time was recorded in seconds using a stopwatch by one experienced investigator. Participants were allowed two trials for each sprinting distance, and the best time was used for analysis.

### 2.7. Agility

Agility was evaluated through the *t*-test. The *t*-test is a four-directional agility and body control test that assesses the ability to change direction rapidly while maintaining balance and without losing speed (Semenick, 1990). Participants sprinted 9.14 m straight, then shuffled 4.75 m to the left side. Next, participants shuffled to the right side 9.14 m and immediately shuffled 4.75 m back. Finally, participants ran backward until they passed the starting point. Test time was recorded in seconds using a stopwatch by one experienced investigator. The best time was retained for analysis.

All fitness test assessments were conducted by trained staff from the investigation team, who were familiar with each protocol.

### 2.8. Statistics

Descriptive statistics are presented as means ± standard deviations. A one-way between-groups analysis of variance (ANOVA) was conducted to explore differences in CA, body composition, and fitness test results between age groups. The Pearson product–moment correlation was used to examine the relationship between CA, body composition, lower-body explosive strength, speed, and agility. Hierarchical multiple regression analyses were conducted to investigate the amount of variance in speed and agility tests explained by the SJ and the CMJ (entered in step 3), after controlling for CA (entered in step 1), and body composition variables (entered in step 2). All analyses were performed using IBM SPSS Statistics software 26.0 (SPSS Inc., Chicago, IL, USA). The significance level was set at *p* ≤ 0.05.

## 3. Results

Table 1 summarizes the descriptive statistics for CA, anthropometry, and fitness tests according to the age group. Results of ANOVA comparing groups are also presented in Table 1. The U19 players were heavier, taller, and had lower BF% than their peers. Overall, the U19 group showed better scores in the fitness tests, except for the 10 m linear sprint time.

Significant results of Pearson product–moment correlations according to each age group are presented in Table 2. Among the U15 group, CA presented significant positive correlations with body mass and lower-body explosive strength. In the U17 group, CA showed relationships with body mass (*r* = 0.29, *p* ≤ 0.05), SJ (*r* = 0.25, *p* ≤ 0.05) and the 35 m linear sprint (*r* = −0.31, *p* ≤ 0.05). In the U19 group, CA was correlated with the SJ (*r* = −0.43, *p* ≤ 0.01), CMJ (*r* = −0.40, *p* ≤ 0.01), and *t*-test (*r* = 0.35, *p* ≤ 0.05). BF% was significantly and positively correlated with body mass, speed, and agility in all age groups. In contrast, BF% was a significant negative predictor of lower-body explosive strength, particularly in the U15 (SJ: *r* = −0.69, *p* ≤ 0.01; CMJ: *r* = −0.63, *p* ≤ 0.01) and U17 (SJ: *r* = −0.28, *p* ≤ 0.05) groups. Overall, linear sprint and *t*-test times were strongly and negatively correlated with lower-body explosive strength. However, the strength of the SJ and CMJ correlations decreased when CA was increased. A significant and positive relationship was observed between linear sprint times and the *t*-test performance in all age groups.

Table 3 describes the results of hierarchical multiple regression analysis conducted to investigate the effects of lower-body explosive strength (entered in step 3) on speed and agility performance, after controlling for body composition (entered in step 2) and CA (entered in step 1). CA and body composition variables explained 43% of the variance observed in the 35 m linear sprint time. The introduction of lower-body explosive strength tests explained an additional 12% of the variance observed in the 35 m linear sprint. The SJ remained the most powerful predictor of the whole model (β = −0.37; *p* ≤ 0.05). Regarding the *t*-test, CA and body composition variables could explain 30% of the variance observed. The addition of lower-body explosive strength tests as predictors increased the ability to explain the variance observed by 17%. The SJ remained the most significant predictor of the *t*-test time (β = −0.36; *p* ≤ 0.05).

## 4. Discussion

Our results indicate that lower-body explosive strength tests strongly correlate with speed and agility performance in the U15, U17, and U19 age groups. After controlling for CA and body composition variables, the SJ remained the most powerful predictor of the variance observed in the linear sprint (10 and 35 m) and *t*-test times.

BF% showed moderate positive relationships with speed and agility in all age groups. Indeed, increased BF% is related to increased time spent during sprinting and change of direction tests [29,30,31]. Besides, significant correlations (negative) were found between BF% and lower-body strength tasks in all age groups. According to the literature, the adverse effect of BF% on sports performance has been apparent in tasks requiring projection and rapid movement, such as running or jumping [32,33]. Among 50 adults involved in at least six months of systematic lower-body resistance training, authors reported that BF% could explain more variability in CMJ than other body composition variables [20]. In football, a study conducted to compare anthropometry and functional capacities among male players from different competitive levels showed a superior ability in sprinting and vertical jumping, and lower BF%, in elite players compared with their peers [30].

Moreover, according to our analyses, the number of relationships between BF% and the fitness tests decreased when CA increased. The results reinforce BF% as a crucial influence on functional capacities, particularly in the younger age groups. Thus, sports agents and coaches involved in youth football should promote multidisciplinary approaches based on specific training and healthy nutritional habits to improve explosive strength and avoid detrimental effects of BF% in sports performance.

Meanwhile, speed and agility performance were strongly and negatively correlated with lower-body explosive strength in all age groups. Indeed, vertical jumping has been consistently associated with speed and agility, since higher levels of lower-body explosive strength are related to lower time spent in sprinting and change of direction tests [3]. Research on the effects of lower-body strength training in adult footballers have revealed a significant relationship between increased back squat and squat strength performance and changes in 5- (*r* = 0.62), 10- (*r* = 0.78), and 20-m (*r* = 0.60) sprint times [12]. Heavy resistance training has been recommended to enhance strength, speed, and agility among elite male footballers [34].

In this study, the correlation between lower-body explosive strength, speed, and agility decreased as CA increased. In the U15 group, both SJ and CMJ presented large relationships with the *t*-test, while in the U19 group, the strength of the relationship decreased to moderate. The same trend was observed for the 10- and 35-m linear sprint. Past research on adolescent male football players from six age categories (from U13 to U19 age groups) showed very large correlations between the CMJ and linear sprints in the U13 and U14 groups. The strength of the correlation decreased to large among the U15 and U16 groups and to moderate or small in the older groups [35]. In interpreting these results, it is crucial to consider the possible effects of growth and biological maturation. In boys, a dramatic improvement of strength and power occurs between 14 and 16 years during the peak height velocity (PHV) [36], which could lead to substantial differences in fitness tests results according to players’ maturity status [33].

After controlling for CA and body composition as possible confounders, the hierarchical multiple regression analyses were used to assess the influence of lower-body strength tasks on speed and agility capacities. The results indicated that the SJ was the most significant predictor of the whole model, accounting individually for 36 to 37% of the variance observed in the *t*-test performance and the 35 m linear sprint. Indeed, the SJ appears to be a powerful tool for assessing lower-body explosive strength. While the CMJ allows for the evaluation of the capability to produce force in stretch–shortening cycle movements quickly, the SJ assesses the ability to rapidly develop force exclusively during the concentric movement [37]. In a study conducted to verify differences in the SJ performed by starter and non-starter Division I female football players, authors concluded that starters exhibited superior height, greater mean velocity, and greater peak velocity during the SJ performance compared to non-starters [38]. Therefore, since the development of lower-body explosive strength is linked with essential attributes of high-quality sports performance such as speed and agility [4,6], specific strength and conditioning programs to improve explosive strength should be considered during the season. Additionally, the SJ may be a simple and powerful tool to assist sports agents and coaches in monitoring and manipulating training methods to optimize lower-body explosive strength.

The current study presents some limitations. We acknowledge some limitations related to accuracy and evaluator dependence of the anthropometric measures. However, these methodologies involve portable and relatively inexpensive instruments. In addition, the procedures were noninvasive, and the assessments were less time-consuming and recognized as reliable and valid to predict total and regional body composition [39]. On the other hand, assessment of the players’ maturity status would allow greater insights into the possible influence of biological variables on anthropometry and fitness test performance. Additionally, the division of the sample into one-year cohorts instead of a two-year span would probably permit the analysis of more balanced groups in terms of individual characteristics. According to the past literature, differences in vertical jumping performance have been mainly reported between larger age intervals (≥2 years) [21,40]. Therefore, future work should consider participants’ maturity status and a narrow age range to be more informative. It may be worth performing a study similar to ours on adult footballers.

This study emphasizes the crucial role of lower-body explosive strength on speed and agility among adolescent male football players, after controlling for CA and body composition. Sports agents and coaches should consider in-season programs focused on lower-body explosive strength development in the youth football training process to improve maximal sprints and change of direction performance. This strategy will allow for the development of youngsters’ physical attributes, enhance game performance, and create pathways to long-term success.

## 5. Conclusions

Our study indicates that lower-body explosive strength, particularly the SJ, is a significant predictor of male adolescent football players’ speed and agility capacities. The SJ test provides insights into players’ ability to produce force during the concentric movement. As an easy and reliable field test to use, SJ may be a powerful tool to assist sports agents and coaches during training monitoring. On the other hand, our results show the strong detrimental relationships between BF%, lower-body explosive strength, speed, and agility. Therefore, it is recommended that multidisciplinary approaches consider a healthy diet and specific strength and conditioning interventions to enhance explosive strength during the season. These strategies will contribute to players’ physical development and improve game performance.

## Figures and Tables

**Table 1:** Descriptive statistics for CA, anthropometry, and fitness tests according to the age group, and results of ANOVA comparing groups.

Variable	Mean ± SD			ANOVA		Post-Hoc Comparisons
	U19 (n = 51)	U17 (n = 62)	U15 (n = 51)	*F*	*p*	
CA (years)	17.8 ± 1.1	15.9 ± 0.6	14.0 ± 0.6	338.906	≤0.01	U19 > U17; U17 > U15
Body mass (kg)	68.8 ± 6.5	64.5 ± 9.1	56.9 ± 10.5	19.905	≤0.01	U19 > U17; U17 > U15
Height (cm)	174.8 ± 6.1	172.0 ± 7.3	165.5 ± 9.3	21.301	≤0.01	U19 > U17; U17 > U15
Body fat (%)	14.1 ± 2.3	16.4 ± 2.2	19.7 ± 2.9	72.937	≤0.01	U19 < U17; U17 < U15
Handgrip (kg)	37.5 ± 6.3	34.2 ± 6.1	28.5 ± 6.8	26.827	≤0.01	U19 > U17; U17 > U15
Sit-ups (n)	22.8 ± 3.1	22.6 ± 3.4	22.3 ± 4.5	0.264	0.768	
SJ height (cm)	31.5 ± 4.9	29.2 ± 3.9	25.3 ± 5.8	25.677	≤0.01	U19 > U17; U17 > U15
CMJ height (cm)	32.2 ± 5.0	29.5 ± 4.1	25.9 ± 4.9	26.321	≤0.01	U19 > U17; U17 > U15
10 m linear sprint (s)	1.79 ± 0.23	1.70 ± 0.20	1.91 ± 0.18	13.577	≤0.01	U17 < U19, U19 < U15
35 m linear sprint (s)	4.83 ± 0.25	4.94 ± 0.34	5.46 ± 0.45	46.574	≤0.01	U19 < U17; U17 < U15
*t*-test (s)	9.60 ± 0.44	10.12 ± 0.53	10.34 ± 0.81	20.336	≤0.01	U19 < U17; U17 < U15

SD (standard deviation); CA (chronological age); SJ (squat jump); CMJ (countermovement jump).

**Table 2:** Significant correlation coefficients for body composition, speed, and agility tests, according to the age group.

Age Group	Variable †	1.	2.	3.	4.	5.	6.	7.	8.
U15	1. CA		0.46 **		0.59 **	0.55 **	−0.37 **	−0.46 **	−0.56 **
	2. Body mass			0.32 *					
	3. BF%				−0.69 **	−0.63 **	0.32 *	0.50 **	0.59 **
	4. SJ					0.93 **		−0.65 **	−0.69 **
	5. CMJ						−0.47 **	−0.64 **	−0.65 **
	6. 10 m linear sprint							0.82 **	0.51 **
	7. 35 m linear sprint								0.77 **
	8. *t*-test								
U17	1. CA		0.26 *		0.25 *			−0.31 *	
	2. Body mass			0.42 **					
	3. BF%				−0.28 *				
	4. SJ					0.87 **	−0.46 **	−0.57 **	−0.59 **
	5. CMJ						−0.35 **	−0.58 **	−0.57 **
	6. 10 m linear sprint							0.81 **	0.32 *
	7. 35 m linear sprint								0.50 **
	8. *t*-test								
U19	1. CA				−0.43 **	−0.40 **			0.35 *
	2. Body mass			0.50 **			0.35 *	0.35 *	
	3. BF%							0.37 **	
	4. SJ					0.89 **		−0.38 **	−0.46 **
	5. CMJ							−0.29 *	−0.42 **
	6. 10 m linear sprint							0.56 **	0.31 *
	7. 35 m linear sprint								0.53 **
	8. *t*-test								

CA (chronological age); BF% (body fat percentage); SJ (squat jump); CMJ (countermovement jump); † The numbers in the columns match the numbers in the rows, identifying each variable; * *p* ≤ 0.05, ** *p* ≤ 0.01.

**Table 3:** Summary of hierarchical regression analysis with CA, body composition, and lower-body explosive strength predicting sprints and *t*-test times.

Variable	10 m Linear Sprint			35 m Linear Sprint			*t*-Test		
	Model I	Model II	Model III	Model I	Model II	Model III	Model I	Model II	Model III
	β	β	β	β	β	β	β	β	β
CA	−0.22 **	−0.06	−0.05	−0.58 **	−0.16	−0.17	−0.46 **	−0.03	−0.03
Body mass		−0.07	−0.01		−0.23 **	−0.14 *		−0.19 *	−0.07
BF%		0.18	0.06		0.44 **	0.19 *		0.48 **	0.19
SJ height			−0.09			−0.37 *			−0.36 *
CMJ height			−0.17			−0.08			−0.17
R^2^	0.05	0.06	0.10	0.34	0.43	0.55	0.21	0.30	0.47
*F* for change in R^2^	8.093 **	3.535 *	3.633 **	83.437 **	40.171 **	38.463 **	42.670 **	23.813 **	29.355 **

Model I: CA, Model II: CA, body mass, and BF%; Model III: CA, body mass, and BF%, SJ height and CMJ height; CA (chronological age); BF% (body fat percentage); SJ (squat jump); CMJ (countermovement jump); * *p* ≤ 0.05; ** *p* ≤ 0.01.

## Data Availability

The data presented in this study are available upon request from the corresponding author.
